# Microbial effects of livestock manure fertilization on freshwater aquaculture ponds rearing tilapia (*Oreochromis shiranus*) and North African catfish (*Clarias gariepinus*)

**DOI:** 10.1002/mbo3.716

**Published:** 2018-08-31

**Authors:** Jeremiah J. Minich, Qiyun Zhu, Zhenjiang Zech Xu, Amnon Amir, Maxon Ngochera, Moses Simwaka, Eric E. Allen, Hastings Zidana, Rob Knight

**Affiliations:** ^1^ Marine Biology Research Division Scripps Institution of Oceanography University of California San Diego La Jolla California; ^2^ Department of Pediatrics University of California San Diego La Jolla California; ^3^ Department of Fisheries Fisheries Research Unit Monkey Bay Malawi; ^4^ Center for Microbiome Innovation Jacobs School of Engineering University of California San Diego La Jolla California; ^5^ National Aquaculture Center Domasi Malawi; ^6^ Department of Computer Science and Engineering University of California San Diego La Jolla California

**Keywords:** 16S rRNA, 18S rRNA, African catfish, antibiotic resistance genes, aquaculture, fish microbiome, freshwater ecology, metagenomics, microbiome, tilapia

## Abstract

The majority of seafood is farmed, with most finfish coming from freshwater ponds. Ponds are often fertilized to promote microbial productivity as a natural feed source to fish. To understand if pond fertilization with livestock manure induces a probiotic or prebiotic effect, we communally reared tilapia (*Oreochromis shiranus*), and North African catfish (*Clarias gariepinus*), for 4 weeks under seven manure treatments including layer chicken, broiler chicken, guinea fowl, quail, pig, cow, vs. commercial feed to evaluate microbial community dynamics of the manure, pond water, and fish feces using 16S and 18S rRNA marker genes along with metagenome sequencing. Catfish growth, but not tilapia, was positively associated with plankton abundance (*p* = 0.0006, *R* ^2^ = 0.4887) and greatest in ponds fertilized with quail manure (ANOVA, *p* < 0.05). Manure was unique and influenced the 16S microbiome in pond water, tilapia gut, and catfish gut and 18S community in pond water and catfish guts (PERMANOVA, *p* = 0.001). On average, 18.5%, 18.6%, and 45.3% of manure bacteria sOTUs, (sub‐operational taxonomic units), were present in the water column, catfish feces, and tilapia feces which comprised 3.7%, 12.8%, and 10.9% of the total microbial richness of the communities, respectively. Antibiotic resistance genes were highest in the manure and water samples followed by tilapia feces and lowest in catfish feces (*p* < 0.0001). In this study, we demonstrate how the bacterial and eukaryotic microbial composition of fish ponds are influenced by specific livestock manure inputs and that the gut microbiome of tilapi*a* is more sensitive and responsive than catfish to these changes. We conclude that animal manure used as fertilizer induces a primarily prebiotic effect on the pond ecosystem rather than a direct probiotic effect on fish.

## 1 INTRODUCTION

Aquaculture is required to feed the world in the 21st century, while doing so sustainably is required to preserve ecosystems. In 2014, for the first time in history, the amount of seafood consumed from aquaculture farms surpassed seafood harvested from the wild (http://www.fao.org/3/a-i5555e.pdf). Fish farming makes up 66% of all aquaculture production with 87% of production occurring in freshwater systems as opposed to marine (Moffitt & Cajas‐Cano, 2014). Across continents, aquaculture is growing fastest in Africa at 11.7% annual growth, thus improving production for fish farmers while ensuring seafood safety for human consumption is needed. With over 354 species of fish being farmed globally, tilapia and catfish species are grown worldwide in over 135 countries and are considered the fastest growing markets making up a large proportion of the total farmed fish biomass (Moffitt & Cajas‐Cano, 2014). In many developing countries, particularly throughout Africa, the aquaculture industry is growing to meet fish consumption demands, while providing numerous job opportunities. Malawi, a country with over 17 million people, was designated the poorest country in the world by the World Bank in 2015, while fishery‐related activities were estimated to provide jobs to 450,000 people and to account for 4% of the nation's GDP (Munthali, 1997). Annual per capita fish consumption in Malawi, however, still remains low at 4.6 kg making Malawi one of the lowest in Africa (Moffitt & Cajas‐Cano, 2014). Lake Malawi, which was estimated to harbor over 1,000 species of fish (Snoeks, 2000), has experienced a steady decline in wild caught, high value tilapia species with an increase in low value sardine species, *Engraulicypris sardella*, which is thought to be due to overfishing and ecosystem degradation through habitat loss (Jamu, Banda, Njaya, & Hecky, 2011). Aquaculture currently comprises approximately 5% of the total fish production in Malawi but has been estimated to have grown 300% in the past 10 years. These efforts are helping to alleviate malnutrition by increasing total fish protein consumption while providing sustainable economic enterprises into the future (Moffitt & Cajas‐Cano, 2014). Determining best practices to promote safe aquaculture while ensuring natural ecosystem conservation is an important concern in Sub‐Sahara Africa where government resource management is often limited by financial resources.

Freshwater fish ponds are often comprised of multiple species of fish reared together to most efficiently utilize feed and habitat. Many countries across Asia and Africa employ these methods to polyculture fish such as tilapia, carp, and catfish which have varied feeding strategies including habitat depth (surface vs. benthic) and ecology (herbivory, omnivory, and or carnivory) (Hofer, 1998;.; Willoughby & Tweddle, 1978). Increasing the genetic diversity through polyculture of multiple carp varieties led to increased growth rates (Moav & Wohlfarth, 1974). Almost 90% of all aquaculture species in Malawi is comprised of the indigenous tilapia, *Oreochromis shiranus*, followed by the red breasted tilapia, *Coptodon rendalli* at 5%, and the African catfish, *Clarias gariepinus,* at 3% (Russell, Grötz, Kriesemer, & Pemsl, 2008). Only one commercial fish feed is available in Malawi (Maldeco starter feed, Maldeco Fisheries, Press Corporation PLC, Mangochi, Malawi), which is a hard pressed sinking feed with various pellet sizes, although little to no published research exists on effects of tilapia or catfish performance. Tilapia and catfish are primarily reared together in earthen ponds which are fertilized weekly or monthly with fresh chicken manure, a natural, low cost biofertilizer, providing nutrients for the growth of plankton. In Malawi, best practices for stocking densities for farmers are at 3 fish per m^2^ or m^3^ with all ponds being no deeper than 1 m while other research has used densities of 2–3 fish per m^2^ (Kang'ombe, Brown, & Halfyard, 2006; Mataka & Kang'ombe, 2007). Chicken manure has been shown to be more efficient than chemical fertilizer at promoting growth of phytoplankton in ponds (Boyd, 1982). Livestock manures promote various zooplankton species populations depending on the animal source with chicken manure out performing both cow, pig, and no manure groups in terms of fish production of *C. rendalli* (Kang'ombe et al., 2006). Probiotic research in aquaculture has demonstrated how bacteria and other microbes can benefit fish health by maintaining water quality, producing antimicrobials to reduce mucosal diseases, and contributing enzymes to digestion of food (Balcázar et al., 2006; Lazado & Caipang, 2014; Wang, Ran, Ringø, & Zhou, 2017). By evaluating the microbial ecology of fish ponds, we aim to understand if the livestock manure has a primarily probiotic or prebiotic effect on the pond water and fish. We further aim to discover specific microbial species which are associated with positive fish growth in the most important aquaculture species in Malawi, tilapia, and catfish.

While fertilizing fish ponds with livestock manure has been shown to have positive impacts on tilapia production (Kang'ombe et al., 2006), it is important to evaluate potential public health and environmental health concerns on fish consumption and pond effluent discharge. Fish is often the primary protein source in developing countries and has been touted as a way to improve nutrition in poor communities (Kawarazuka & Béné, 2011). Reducing malnutrition by increasing protein consumption and household income is a proven, effective strategy in developing countries. In several Sub‐Saharan African countries, animal food consumption was higher in children living with dairy cows or chickens (Hetherington, Wiethoelter, Negin, & Mor, 2017). While living close to animals can improve protein consumption, increased exposure to disease and antibiotic resistance genes are of concern as some microbes associated with livestock can impact human health. Use of antibiotics in livestock production has led to an emergence of antibiotic resistant bacteria (Davies & Davies, 2010; Su, Cui, Chen, An, & Zhu, 2017). Antibiotic resistant bacteria can be found in animal intestinal tissue (Su et al., 2017) and can be transmitted to humans through manure. These antibiotic resistance genes can spread from livestock to humans through human consumption of crops contaminated with infected green manure fertilizer (Heuer, Schmitt, & Smalla, 2011; Thanner, Drissner, & Walsh, 2016) or composted manure fertilizer especially if the livestock is fed antibiotics (Zhu et al., 2013). Although antibiotics are rarely used directly in aquaculture in Malawi, they are used in terrestrial livestock agriculture and further 80% of these antimicrobials remain active in aquaculture water systems (Cabello et al., 2013). Thus, understanding the microbial ecology and transmission of livestock manure microbes in a freshwater pond setting will provide context for human exposure risks to water and fish from these environments.

To understand the mechanisms for the beneficial effects of livestock manure fertilization in aquaculture ponds and transmission impacts, we evaluated the fish pond microbiome using traditional marker gene amplicon methods and untargeted shotgun metagenomics. Over 4 weeks, tilapia and catfish were commonly reared in 4 m^2^ concrete ponds receiving either commercial feed or fresh manure fertilizer in triplicate sourced from layer chicken, broiler chicken, cow, pig, guinea fowl, or quail and applied at 500 kg HA^−1^ week^−1^ beginning 2 weeks prior to fish stocking. For the no manure tank, fish were fed with Maldeco starter fish feed at 10% bodyweight day^−1^ (Maldeco Fisheries, Press Corporation PLC, Mangochi, Malawi). At the end of 4 weeks, water quality and fish performance was assessed. A total of 127 samples from pond water, tilapia feces, and catfish feces were DNA extracted and analyzed using 16S rRNA (96), 18S rRNA (127), and community metagenome sequencing (96) to assess microbiome dynamics.

## 2 METHODS

### 2.1 Experimental design

#### 2.1.1 Tank setup

At the National Aquaculture Center in Domasi, Malawi, 21 outdoor concrete tanks (2 m × 2 m × 1 m) were thoroughly cleaned, filled with fresh water from the Domasi River. The water levels were maintained at 1 m throughout the experiment to combat evaporation, but were not replenished so as to mimic standard aquaculture pond practices. Livestock manures including layer chicken, broiler chicken, quail, guinea fowl, pig, and cow were sourced at the beginning of the experiment from local farms and stored in plastic bags in the dark for the duration of the 6‐week experiment. To initialize development of microbial biomass (e.g., algal bloom), tanks (*n* = 18) were fertilized in triplicate with one of the six livestock manures for 2 weeks at the standard rate of 500 kg HA^−1^ week^−1^. At the 2‐week mark (week 0), each tank was stocked with four catfish and six tilapia (2.5 fish/m^2^) at 2 and 4 months of age, respectively. Smaller, younger catfish were used to minimize predation on tilapia. For the next 4 weeks, tanks were continually fertilized at (500 kg HA^−1^ week^−1^). For a positive control (*n* = 3 tanks), which we expected maximum fish growth, we substituted manure for the only commercially available fish feed in Malawi, Maldeco starter feed, with a pellet size of 2 mm and advertised crude protein content of 25%. In these tanks, fish were fed at 10% body weight per day, twice daily. Fish were previously conditioned by a 3 day fast. Fish mass and total lengths were taken at the beginning (week 0) and end of the experiment (week 4) with total biomass per species being equal across tanks. Unfortunately, due to lack of available catfish fingerlings, the total biomass of the four catfish replicates in the Maldeco feed tank was slightly larger (one‐way ANOVA, *p* > 0.05) than the other tanks, so Maldeco feed growth rates were excluded from comparisons of growth in the analyses. As a reference, however, the bar charts are still included in the figures. Mortalities were monitored and counted for each tank. Fish were collected from the tanks with mass and length measured. A secchi‐disk was used to measure water visibility at the end of the experiment. The depth at which the disk is no longer visible was recorded and is related to turbidity influenced by primary productivity, fish feces, and detritus (Almazan & Boyd, 1978).

### 2.2 Fish performance

The following equations were used to measure fish performance over the course of the experiment. For species specific performance, survival rate, average daily growth, specific growth rate, and percent weight gain was compared using an unpaired student's *t* test. Fish growth and performance was tested for normality and then compared across fertilization treatments by a one‐way ANOVA and Tukey's post hoc test if significant. Mortalities were determined by daily monitoring of dead fish on the surface. Fish mortalities due to cannibalism or carnivory were determined by counting the total fish at the end of the experiment and subtracting known mortalities during the 4 weeks.

To measure the total change in biomass of a specific fish species over the course of the treatment:

*Percent Weight Gain* (Hopkins, 1992; Lugert, Thaller, Tetens, Schulz, & Krieter, 2016)

$$\text{(PWG)}=\left(W_{t}-W_{i}\right)/W_{i}\times 100$$

*W*_t_ = total mass (grams) at end of experiment

*W*_i_ = total mass (grams) at start of experiment

*Average Daily Growth* (Hopkins, 1992)

$$\text{(ADG)}\left(g\;{\text{day}}^{-1}\right)=W_{t}-W_{i}/t_{\mathrm{days}}$$

*t *= number of days

*Specific Growth Rate* (Hopkins, 1992)

$$\text{(SGR)}=100\times \left(\ln W_{f}-\ln W_{i}\right)/t_{\mathrm{days}}$$

*Survival Rate* is measured by (*Q*_f_/*Q*_i_) × 100

*Q*_f_ = quantity of fish at end of experiment

*Q*_i_ = quantity of fish at beginning of experiment

To measure the plumpness of the fish which is an indirect measurement of health:

*Condition Factor* (Froese, 2006; FULTON, 1904; Lugert et al., 2016)

$$\left(K\right)=100\times {\text{mass}}_{\mathrm{grams}}/{\left({\text{length}}_{\mathrm{cm}}\right)}^{3}$$

### 2.3 Microbiome sampling and processing

Water was collected from the 21 ponds at week 2 and week 6 to determine microbial diversity pre‐ and postfish stocking during a fertilization regime. At week 2, 50 ml of water was sampled from the upper 30 cm of pond water from each of the 21 tanks with replicate treatment tanks pooled and then 0.22 μm filtered to collect all microbes (bacteria, phytoplankton, and zooplankton). At the end of week 6 (week 4 with fish), 150 ml of water from each of the individual 21 tanks was collected and 0.22 μm filtered. Fecal samples were collected by stomach massaging from 1 fish of each species per tank for a total of 21 tilapia and 21 catfish samples. In addition, four catfish were processed after the initial fasting as a time zero control. These fish included two adult broodstock and two juveniles. One adult and one juvenile were also processed whole for skin, intestinal, stomach, and fecal samples. Three tilapia were also processed after the initial fasting as a time zero control for fecal samples only. As a control reference for comparing concrete ponds to large earthen ponds, two water samples were also taken from earthen ponds at the National Aquaculture Center (NAC) along with fecal samples from three *Coptodon rendalli* or *Tilapia rendalli*, another commonly cultured tilapia species in Malawi. As an additional control, we collected, isolated, and processed the five primary zooplankton found in Lake Malawi at Senga Bay including the calanoid copepod *Tropodiaptomus cunningtoni*, the cyclopoid copepods *Mesocyclops aequatorialis aequatorialis* & *Thermocyclops neglectus*, and the cladocerans *Diaphanosoma excisum* & *Bosmina longirostris* (Irvine & Waya, 1999). Genomic DNA was extracted from livestock manure input, water filters at time 0 and week 4, tilapia feces, and catfish feces at Chancelor College using the Mobio PowerSoil kit (Carlsbad, USA) following the Earth Microbiome Project protocols (earthmicrobiome.org).

For amplicon methods, the Earth Microbiome Project protocols were followed (Thompson et al., 2017). Briefly, the gDNA was amplified in triplicate for 35 PCR cycles using the 16S 515f/806rB V4 region prokaryote primers (Caporaso et al., 2011; Walters et al., 2016) and broad range 18S 1389/1510 V9 region eukaryote primers (Amaral‐Zettler, McCliment, Ducklow, & Huse, 2009) and sequenced at 2 × 150 bp V3 Illumina chemistry (Caporaso et al., 2011, 2012; Walters et al., 2016). The analysis was performed in Qiita and QIIME 1.9.1 (Caporaso et al., 2010) and visualized in EMPeror (Vázquez‐Baeza, Pirrung, Gonzalez, & Knight, 2013) with PCoA plots generated by calculating unweighted and weighted unifrac distances (Lozupone & Knight, 2005). Finer‐grained de novo sequence analysis was performed by deblur (Amir et al., 2017) and visualized in Calour (github.com/amnona/calour) providing single‐SNP resolution of sOTUs (sub‐operational taxonomic unit). For both 16S and 18S deblurred BIOM tables, only samples with at least 5,000 reads and sOTUs which had at least 100 reads across all samples were included in downstream analysis. Microbial richness was calculated by counting the total observed sOTUs per sample. To determine if microbial richness was associated with fish performance or water column microbial growth, linear regressions of PWG vs. secchi disk visibility and microbial richness vs. secchi disk variability were performed.

For whole genome shotgun (WGS) metagenomics, gDNA was fragmented and made into libraries using the KAPA Hyper Plus kit (F. Hoffmann‐La Roche Ltd, Swiss). Final libraries were size selected at 400–600 bp using a Pippin prep (Sage Science, USA) and sequenced on an Illumina 2 × 150 bp Rapid Run. To remove as much host associated DNA sequences as possible, reads were processed through bowtie2 (Langdon, 2015). For tilapia samples, the closest relative with a genome reference was *O. niloliticus* (MKQE0000000.1) while the nearest phylogenetic catfish reference was *Ictalurus punctatus* (GenBank: LBML00000000.1), followed by trimming of poor quality reads and visualization using Trimmomatic (Bolger, Lohse, & Usadel, 2014) and FASTQC, respectively. Sequences were then assigned taxonomy using the *k*‐mer‐based metagenome profiler Kraken (Wood & Salzberg, 2014). Differential abundances of sOTUs or metagenome associated taxa were determined using a permutation‐based group‐mean comparison with FDR controlled to 0.1 using the Benjamini‐Hochberg procedure within deblur and Calour (Benjamini & Hochberg, 1995). For the analysis of antibiotic resistant genes (ARGs), reads were translated into partial protein sequences using the gene calling program FragGeneScan (Rho, Tang, & Ye, 2010). This was followed by search against the HMM database for antibiotic resistance protein families, ResFams (Gibson, Forsberg, & Dantas, 2015), using HMMer v3.1b2 (Finn, Clements, & Eddy, 2011). AGR abundances were computed as the count of partial protein sequences matching each antibiotic resistance protein family and normalized by total number of reads for each sample.

### 2.4 Effect of manure

To determine which sOTUs from the manure fertilizer may be influencing the gut communities in the fish through direct transmission from ingestion or through the water column, the number of sOTUs present in both manure and the various environments (water week 0, water week 4, tilapia feces,and catfish feces) was determined for each manure type. Any sOTU present in both a manure primary sample and one of the three replicate environment types was considered shared and included. A two‐way ANOVA with multiple comparisons was run to determine if environment types differed in the number of manure sOTUs present and if there was an effect of manure type. This comparison was only performed for 16S and not 18S due to a higher success rate with 16S (86% vs. 55%). Sample drop out did not permit a large enough sample size to perform the calculations on 18S. Guinea fowl was excluded because the week 0 water sample was dropped out. The manure sOTUs present in the tilapia and catfish feces for both 16S and 18S was visualized in a Venn diagram to determine if there was a core set of manure derived microbes shared with either fish. Specifically, core microbes were defined as being present in at least 75% of samples. To determine if livestock manure, when used as a fertilizer in fish ponds, influences the microbial communities (16S, 18S, and WGS) of the water column, tilapia feces, and catfish feces, we performed a PERMANOVA (Anderson, 2001) in QIIME 1.9.1 (Caporaso et al., 2010).

## 3 RESULTS

### 3.1 Fish performance

**Figure 1:**
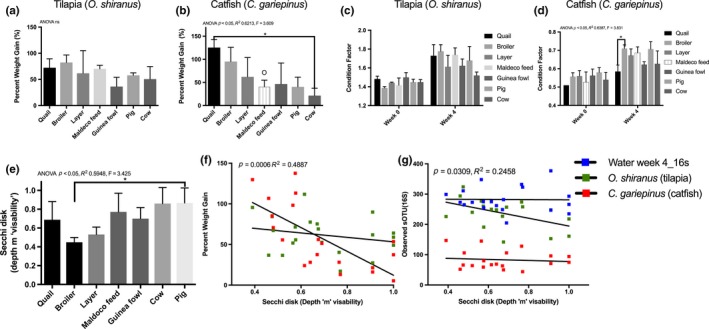
Tilapia and catfish performance metrics during a four week growout experiment under seven fertilization strategies. Tilapia (a) and catfish (b) growth performance according to the fertilization strategy. Condition factor comparisons across fertilization regimes for (c) tilapia and (d) catfish. The influence of fertilization method on water visibility (e) as measured by secchi disk at week 4 was compared. One‐way ANOVA with Tukey's multiple comparisons test was used to compare means by fertilization method across the species specific performances. (f) The PWG of tilapia (green squares) and catfish (red squares) was compared to water visibility using linear regression. (g) The microbial richness or total number of sOTUs found in the fish guts and water column were compared to water visibility which is a proxy for microbial growth. (****p < 0.0001; ***p < 0.001; **p < 0.01; *p < 0.05)

Fish performance as determined by survival rates, growth rates, SGR, ADG, and condition factor differed between the tilapia and catfish which were reared together (Figure 1, Supplementary Table S1). Of all 21 tanks, only five catfish mortalities were recorded and all attributed to carnivory, whereas 43 tilapia died with 49% attributed to carnivory and the rest, natural causes or stress. Across all 21 tanks, catfish (*M*,* SE*; 93.75, 3.08) had a significantly (*p* < 0.0001) higher survival rate than tilapia (66.67, 3.81) with the pig treatment having the highest mortalities with both species. Guinea fowl ponds had the highest survival rates for tilapia followed by broiler and quail although this was not significant. The average daily growth rate in grams per day was higher in tilapia (0.32, 0.03) compared to catfish (0.23, 0.03) (*p* < 0.05) (Supplementary Table S1), whereas specific growth rates (Supplementary Table S1) for tilapia (1.68, 0.11) and catfish (1.54, 0.20) and percent weight gain for tilapia (61.63, 5.08) and catfish (58.47, 9.06) followed the trend with tilapia having higher growth performance values although this was not significant (Supplementary Table S1).

### 3.2 Manure influence on fish performance

The manure fertilizer treatments had a greater influence on catfish performance than tilapia as demonstrated by a greater variability and significance in ANOVA testing (Figure 1a,b). For catfish, quail manure provided the best conditions for growth compared to cow manure while broiler manure was also high (Figure 1b). Although not significant, broiler manure also had the greatest growth performance in tilapia (Figure 1a). For both tilapia and catfish, the condition factor increased from week 0 to week 4 which is expected since the fish were fasting in the beginning. At week 4, manure treatments did not influence the condition factor of tilapia, whereas catfish reared in broiler manure had a higher condition factor than those in quail manure (*p* < 0.05) (Figure 1c,d). Manure treatment influenced the secchi disk visibility across the study (ANOVA *p* < 0.05, *R* ^2^ = 0.5948, *F* = 3.425), with the broiler manure pond having a higher plankton density than the pig manure pond (Figure 1e). Further, high plankton growth (lower water visibility) was correlated with increased percent weight gain in catfish (*p* = 0.0006, *R* ^2^ = 0.4887) but not tilapia (Figure 1f). Plankton growth was not associated with an increased microbial richness in the water column or catfish feces (Figure 1g), but was associated with an increased microbial richness in tilapia feces (*p* = 0.0309, *R* ^2^ = 0.2458) (Figure 1g).

Catfish performance was more responsive than tilapia to fertilizer input and microbial production in the ponds. For tilapia, the PWG was higher, although not significant, in quail and broiler manure tanks compared to the positive control tanks of commercial Maldeco fish feed only (Figure 1a). Tilapia did, however, grow more than catfish in the commercial feed pond.

### 3.3 Microbiome

**Figure 2:**
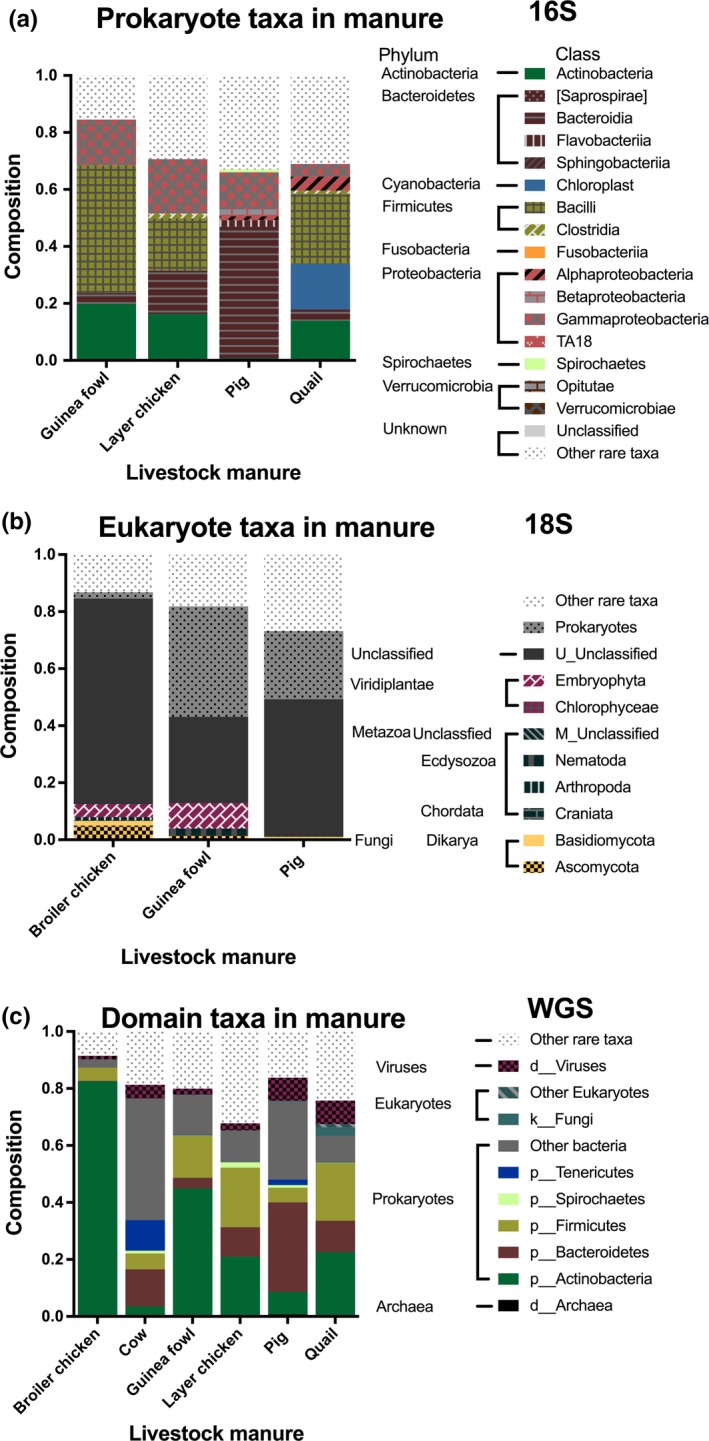
Microbial composition differs across livestock manure. Microbial composition organized by phylogenetic grouping for (a) 16S rRNA, (b) 18S rRNA, and dom (c) k‐mer profile from whole genome sequencing

**Figure 3:**
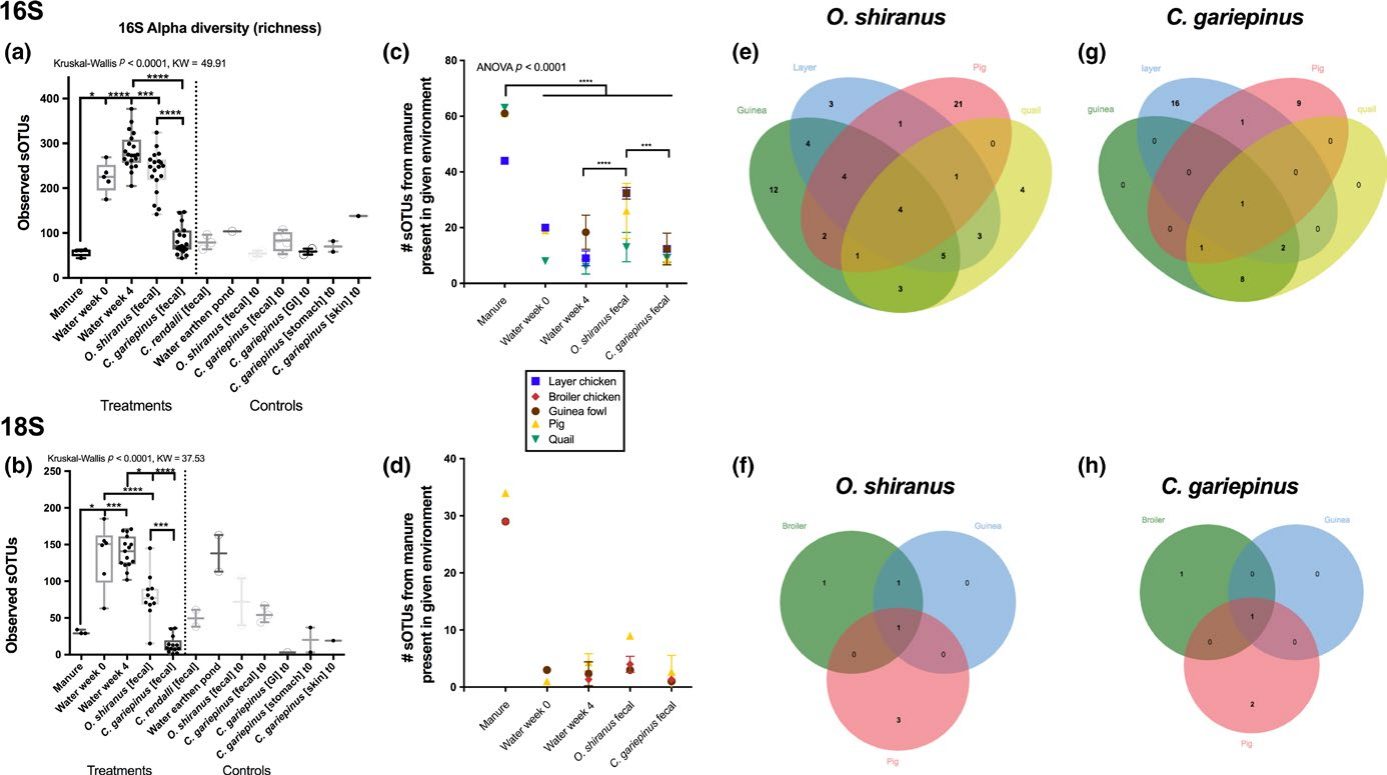
Influence of manure associated microbes on pond ecology. (a) 16S and (b) 18S microbial richness across sample types were compared with non‐parametric Kruskal‐Wallis test with Benjamini‐Hochberg 0.05 FDR. Presence of manure specific sOTUs, (c) 16S and (d) 18S, were counted across the sample types and 16S compared with a two‐way ANOVA with Tukey multiple comparisons test. 18S was not compared due to multiple sample drop out. The core sOTUs shared between manure inputs and fish guts were determined for (e, f) tilapia and (g, h) catfish

There were 83 (of 96) samples for 16S rRNA, 70 (of 127) for 18S rRNA, and 96 (of 96) for whole genome shotgun (WGS) metagenomes which had sufficient reads and were included in the microbiome analysis. There were 826 sOTUs with 16S, 442 sOTUs for 18S, and 898 taxonomic sOTUs for WGS. Manure microbiomes were successfully sequenced for four manure types with 16S, three with 18S, and all six with shotgun metagenomics (Figure 2). The number of observed sOTUs or microbial richness for 16S and 18S differed across environment types (Kruskal‐Wallis *p* < 0.0001) (Figure 3a,b). Microbial richness in manure samples was substantially lower than other environments like water and fish feces. For 16S, both water column time points: week 0, week 4, and tilapia feces had higher microbial richness (3.9‐, 4.9‐, and 4.1‐fold) than the manure samples, while catfish was only 1.5× higher (Figure 3a). For 18S, which evaluates eukaryotic diversity, microbial richness in water at week 0 and week 4 was 4.4‐ and 4.6‐fold higher than manure samples (Figure 3b), while tilapia was only 2.6‐fold higher. Water at week 0 and week 4 had higher richness than tilapia and catfish feces while tilapia had a higher number of sOTUs than catfish (Figure 3b).

### 3.4 Manure effect on pond microbiome

Next, we used metagenomic sequencing methods to evaluate if animal manures differed in their microbial diversity and if these differences altered the pond water and fish fecal communities in a specific way. Manure samples from different livestock species had highly variable microbial communities as expected from other microbiome studies. For 16S, bird manure generally had a higher composition of *Actionbacteria* and *Firmicutes*, specifically *Bacilli*, whereas the pig sample was dominated by Bacteroidetes and specifically *Bacteroidia* with minor amounts of *Flavobacteria* (Figure 2a). Quail manure had high amounts of chloroplast reads and elevated composition of *Alphaproteobacteria* (Figure 2a). Although only three of the six manure samples yielded sufficient reads for the 18S analysis, bird samples had higher amounts of plant (Embryophyta) associated reads and fungi associated reads from *Basidiomycota* and *Ascomycota* (Figure 2b). WGS sequencing revealed manure samples having variable types of archaea, bacteria, eukaryotes, and viruses depending on the animal source. Within the bacteria, bird manure, especially broiler chicken, had a high composition of Actinobacteria (Figure 2c). Tenericutes were noticeably higher in the cow and pig manure samples (Figure 2c). While Firmicutes and viruses were present across all samples, they were the lowest in the broiler manure (Figure 2c).

To determine if manure fertilizer treatment influenced the microbial communities of individual sample types (water, tilapia feces, and catfish feces), a PERMANOVA was performed on the 16S and 18S datasets. For unweighted UniFrac distances of 16S, fertilizer type significantly influenced the beta diversity of water at week 4, tilapia feces, and catfish feces (PERMANOVA, *p* = 0.001, see Figure S1). Whereas for unweighted UniFrac 18S distances, fertilizer type influenced the beta diversity of only the water column at week 4 and the catfish feces (PERMANOVA, *p* = 0.001, see Figure S1). Livestock manure type influenced the fish pond microbiomes in a variety of ways, particularly within the bacteria domain (16S); therefore, the next goal was to determine which specific microbial sOTUs, within the communities, were being most influenced.

We next investigated if specific sOTUs from the manure were directly influencing the pond ecosystem. The number of 16S sOTUs in guinea fowl, layer chicken, pig, and quail manure was 61, 44, 61, and 63 respectively. There were a total of 120 unique sOTUs from the manure samples of which 38 were present in at least 75% of the livestock manure samples. The majority of these core manure sOTUs, 33 sOTUs or 86.8%, were not shared across any other environment's core microbiome indicating that the manure contributes very little diversity directly to the water and fish gut communities (see Figure S2). Of the five manure 16S‐derived sOTUs that were present in other environments, *Succinivibrio* was a core microbe in both water time‐points and tilapia. Clostridiaceae SMB53 was in water week 0 and tilapia. *A. lwoffi* in water week 0, and an *Escherichia coli* or *Shigella flexneri* strain was shared with the catfish core microbiome. There were 4 core 18S sOTUs in the manure samples while none of these were detected in the water or fish feces (see Figure S3). The majority of 18S reads in the fish samples were from the host (see Figure S3a), with Rotifera reads mostly present in tilapia and plant based angiosperm reads in the catfish (see Figure S3b). The water column was mostly dominated by *Vampyrella* amoeba, Rhizaria, and Rotifera.

In addition to the core analysis, we also evaluated the frequency of manure sOTUs (16S and 18S) in the various environments (pond water, tilapia feces, and catfish feces) within each specific manure type. The type of manure did not influence how many manure associated 16S or 18S sOTUs were present in the water column or in the fish feces. Water samples, tilapia feces, and catfish feces all had significantly less sOTUs present compared to manure (Tukey HSD, *p* < 0.0001) (Figure 3c). Different environments, however, had varying amounts of manure associated sOTUs (Two‐way ANOVA *p* < 0.0001, *F* = 88.43). Specifically, water at week 4 had less manure derived sOTUs than catfish feces while tilapia feces had more manure derived sOTUs than catfish feces (Tukey HSD, *p* < 0.0001) (Figure 3c). Tilapia feces contained a total of 68 unique sOTUs from the four manure samples, with 41.2% of the sOTUs present in at least one other manure sample (Figure 3e).

Looking more closely at the manure associated bacteria found in the various environments, four bacterial sOTUs were found in all four manure types and were shared with tilapia feces including Vibrionaceae, Clostridiaceae SMB53, *Corynebacterium*, and *Brevibacterium aureum*. Catfish had only 38 unique sOTUs shared with manure with 34.2% shared in at least one other manure sample (Figure 3g). One sOTU, Vibrionaceae, was present in all manure and catfish samples. For both tilapia and catfish, several microbes were shared from unique manure sources. Both fish species had *Lactobacillus* and *E. coli* sOTUs shared with guinea fowl, layer chicken, and quail manure. Lastly, *Acinetobacter* was present in both fish and manure from pig and quail ponds.

The number of 18S sOTUs in broiler chicken, guinea fowl, and pig manure was 29, 29, and 34 sOTUs, respectively (Figure 3d). Only one sOTU from the manure samples were also present in either of the fish species fecal material (Figure 3f,h). Aside from the host derived sOTUs, an Embryophyta plant sOTU was present in tilapia from broiler and guinea fowl treated tanks while a Chlorophyceae was present in pig manure and tilapia.

### 3.5 Microbial community analysis

**Figure 4:**
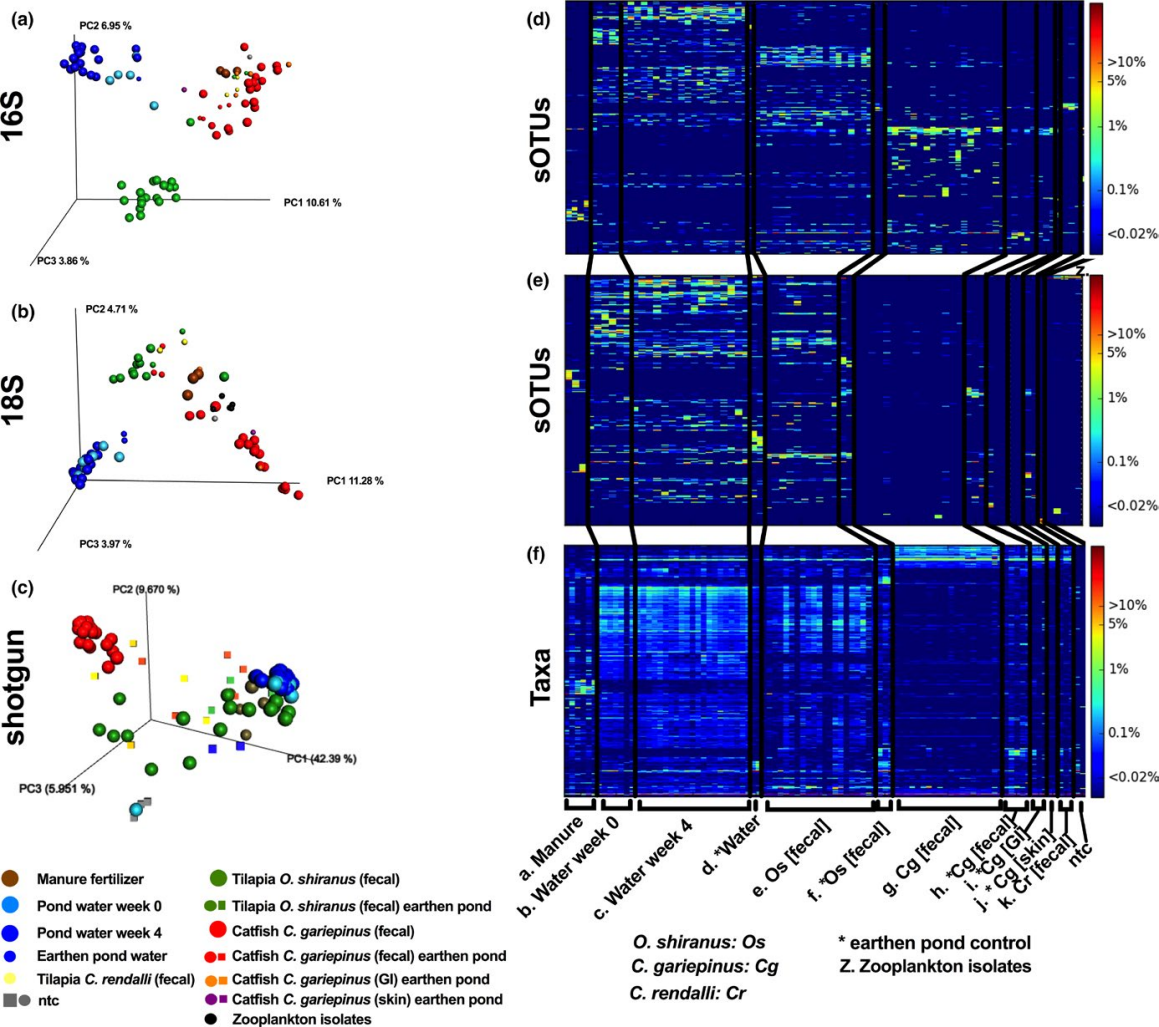
Community level comparison of microbiome associations across sample types in the fish pond system. Principal coordinate analysis (PCoA) plots are based on unweighted (a, b) and weighted (d, e) unifrac distance matrix of the deblurred 16S rDNA amplicon libraries (a, d), deblurred 18S rDNA amplicon libraries (b, e). The shotgun metagenomics is based on jaccard and Bray‐Curtis distances from kraken k‐mer profiling libraries (c, f). Circles represent primary samples from the concrete growout tanks whereas squares are controls from earthen ponds. Heatmap of individual sOTUs *y*‐axis and samples *x*‐axis for (g) 16S, (h) 18S, and (i) shotgun tables are depicted

The sample type (manure, tilapia feces, catfish feces, water week 0, water week 4, and various controls) had the strongest effect on explaining significant differences in the beta diversity of the microbial communities across the samples (PERMANOVA: 16S *n* = 83, 12 groups, *F* = 4.42, *p* = 0.001; 18S *n* = 70, 13 groups, *F* = 7.69, *p *= 0.001; WGS *n* = 96, 12 groups, *F* = 12.19, *p* = 0.001). To evaluate microbial community level similarity in the manure, pond water, tilapia, and catfish fecal microbiomes, the 16S, 18S, and WGS sequencing taxonomies of individual samples summarized using unweighted UniFrac distances are visualized using the principal coordinates analysis (PCoA) (Figure 4a–c). Individual samples and their respective sOTUs were also visualized with a heatmap (Figure 4d–f) depicting several emerging trends. Although manure samples were highly variable with respect to each other, at the community level they form a unique cluster (brown) differentiated from the other sample types (Figure 4a–c). Further, the co‐occurring manure sOTUs were in low proportional abundance in the water column and fish feces (Figure 4d–f). Tilapia and catfish were reared in polyculture, sharing the same exposure to water microbes and food sources, but here we demonstrate the large dissimilarity between the fish (tilapia—green spheres; catfish—red spheres) fecal microbiomes both at the community level (Figure 4a–c) and at the individual sOTU level (Figure 4d–f). The fish fecal microbiomes were also distinct from the water column indicating a unique host associated microbial community although the tilapia feces did share more core microbes with the water column such as Cyanobacteria (Figure 4d–f; see Figure S2a,b). Fish feces shared high compositions of Fusobacteriales (primarily *Cetobacterium somerae*), Clostridiales, Bacillales, and several orders of Proteobacteria including Enterobacteriales, Burkholderiales, and Xanthomonadales (see Figure S2a). Catfish were enriched in Clostridiales, Bacillales, and Lactobacillus within the Firmicutes along with Bacteroidales and Thermales orders (see Figure S2a). Tilapia were enriched with Neisseriales, Rhodobacterales, various Cyanobacteria, and Verrucomicrobia compared to the catfish. When comparing 18S, tilapia feces consistently contained higher proportions of rotifers, while catfish feces were mostly comprised of reads associated with Angiosperms (flowering plant). Lastly, the water column differed slightly at the two time‐points with a shift in microbial composition (Figure 4). There was an increase in Actinobacteria, Saprospirales, and Pirellulales and a decrease in Burkholderiales, Rhodospirillales, and Cyanobacteria 16S sequences between the two water time‐points (see Figure S2a) along with a notable shift in rotifer and cercozoa composition within 18S (see Figure S3a).

### 3.6 Fish microbiomes differ by species

Polycultured tilapia and catfish shared only a few common fish specific fecal microbes, largely having unique host‐specific microbiomes different from manure type. Tilapia had a higher microbial richness than the catfish with a median richness of 248 vs. 71 for 16S (*p* < 0.0001) and 77 vs. 9 for 18S (*p* < 0.001) (Figure 3a,b). The core microbiome community of the tilapia consisted of 125 16S sOTUs while the catfish had only 16 16S sOTUs. Ten of the core catfish microbes were also part of the tilapia core microbiome with seven of these not being shared with the water column or other environment. These seven included two Xanthomonadaceae, one *Chelatococcus*, and three Bacillaceaeae: *Geobacillus*,* Geobacillus thermodenitrificans*, and *Bacillus selenatarsenatis*. Although some microbes were shared, the majority of the microbial composition between the two fish species was different as demonstrated by unique clustering in the PCoA (Figure 4). Tilapia and catfish fecal microbiomes were distinguished by 246 16S (see Figure S4), 65 18S (see Figure S5), and 700 WGS features (see Figure S6) of 826, 472, and 898 total features, respectively. Tilapia were enriched in many Cyanobacteria, Pirellulales, green algae, and other phytoplankton microbes while catfish was mostly distinguishable by its bacteria including several Lactobacilli, Xanthomonadacea, Mycoplasmataceae, and numerous viruses (see Figure S3b, Figure S4b, Figure S5b). The catfish 18S gene represented 98.51% of the composition of the 18S genes with few fungi and plant material making up the remainder. Tilapia and catfish reared in the same pond had species specific fecal microbiomes indicating external components such as diet preference or niche, pond habitat type, or genetics driving the microbiomes.

**Figure 5:**
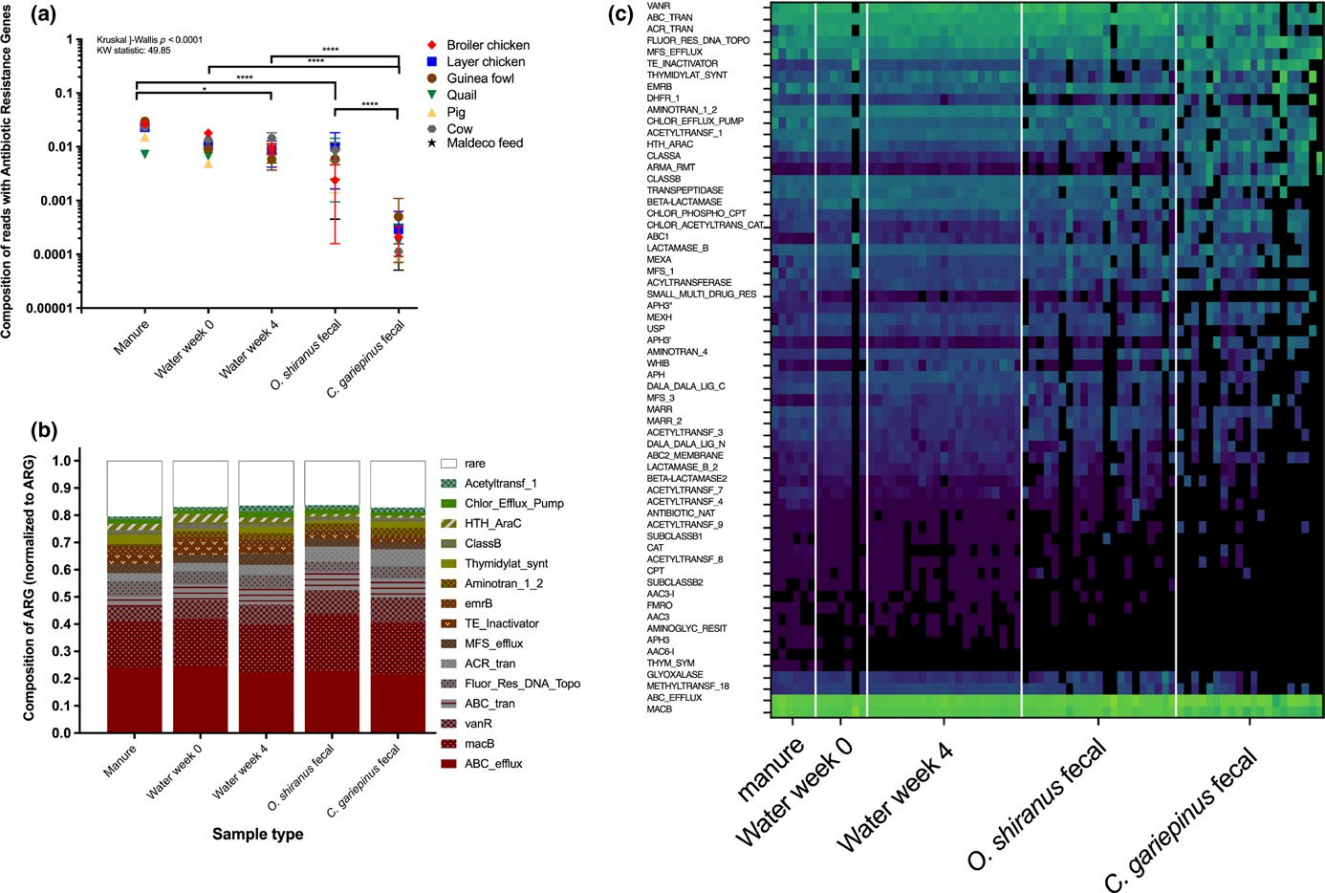
Prevalence of antibiotic resistance genes (ARGs) within the fish pond ecosystem. (a) Total number of ARGs detected across sample types colored by type of manure used as fertilizer. Nonparametric Kruskal‐Wallis testing with post hoc multiple comparisons (Benjamini‐Hochberg FDR) was performed on total ARG composition in metagenomes across sample types. (b) Heatmap depicting absolute ARG composition in metagenomes across all samples grouped by sample type and ordered by manure types: broiler, cow, guinea fowl, layer, maldeco feed, pig, quail (green—high, blue low). (c) Top 15 ARGs averaged across sample types

The proportion of ARGs varied across the five sample types: manure, water week 0, water week 4, tilapia feces, and catfish feces (Kruskal‐Wallis *p* < 0.0001, KW statistics = 49.85). Specifically, ARGs were highest in manure samples (representing 0.7%–2.96% of reads) followed by water week 0 (0.4%–1.8%), water week 4 (0.58%–1.4%), tilapia feces (0.24%–0.99%), and catfish with the least (0.008%–0.05%) (Figure 5a). Of the 166 ARG families in the Resfams database, there were 61 ARGs detected in the fish pond dataset (Figure 5b). When averaging the compositions of individual ARGs within each sample type, the top five ARGs were three ABC transporters (ABC efflux pump, ABC_tran, and macB), one Glycopeptide resistance (vanR), and one Quinolone resistance (Fluor_Res_DNA_Topo), making up 55%–63% of all ARGs (Figure 5c). Tanks that did not receive any animal manure input, but instead were given fish feed, had the same levels of ARGs in the water and fish feces.

## 4 DISCUSSION

Globally, finfish aquaculture production, which is highest in China and India, is primarily performed in freshwater open pond systems (Zhao & Shen, 2016) where livestock manure is often used to fertilize ponds “integrated agriculture systems” (Prein, 2002), providing a low cost feed alternative but posing potential threats to human health (Sapkota et al., 2008; Xiong et al., 2015). We evaluate the mechanisms by which seven types of fertilizer input influences the free‐living and host associated microbial communities of ponds. Specifically, we use high‐throughput sequencing of three types of metagenomic endpoints (16S, 18S, and whole genome sequencing) to evaluate how bacterial and eukaryote microbial communities of pond water and polycultured tilapia and Africa catfish respond to fertilizer treatment. Tilapia and catfish grew most in the broiler chicken and quail manure treatments although the catfish overall growth response was greatest to varying manure treatments. Livestock manure microbiome composition differed depending on the host species source and significantly influenced the water column and fish gut microbiomes. Tilapia gut microbiomes were more affected by these changes than catfish, with the catfish microbiome being more stable. The majority of manure microbes were not detected in the water column or fish guts suggesting a primarily prebiotic mechanism on pond communities by way of contributing organic matter rather than living microbes to the system. Our results provide one of the first experimental microbiome studies (Ghanbari, Kneifel, & Domig, 2015; Tarnecki, Burgos, Ray, & Arias, 2017) on two different fish species grown in polyculture and provides evidence to suggest manure having a primarily prebiotic effect on a pond ecosystem.

Growing fish in polyculture is an applied ecological technique to increase sustainability by improving feed and water utilization. The focus of this study was to evaluate how the microbial community of fish ponds changed with fertilization type. Fish performance was measured and related to the water the fish gut microbiome, but should be scaled to larger sample sizes and ponds in the future to follow up on findings. Fish growth varied across manure treatments and was species specific although both tilapia and catfish grew the most in the broiler chicken and quail manure treatments. Tilapia growth, however, was more variable across replicate tanks whereas catfish growth was less variable and more differentiated. This is surprising because tilapia are generally considered to occupy a lower trophic level as they are omnivorous and herbivorous filter feeders while catfish are generally carnivorous in the wild. Indeed, tilapia feces had higher microbial richness and higher frequencies of manure associated sOTUs compared to catfish including. It is possible that the differences in growth rates could also be attributed to the fact that the fish ages of tilapia and catfish differed at the beginning of the experiment with catfish being younger. As catfish growth was strongly associated with pond primary productivity, the fish may directly benefit by decreased visibility or light intensity (Hossain, Beveridge, & Haylor, 1998), increased sedimentation from microbial turnover, or free living plankton consumption from the water column or benthic (Bok & Jongbloed, 1984). While catfish fry, recent hatchlings, are known to have high survival rates and increased growth with live zooplankton feed, juvenile, or adult catfish may also benefit from green water microorganisms (Neori, 2011). Catfish growth and survival is also improved with lower light penetration or shade in ponds (Appelbaum & McGeer, 1998) which would also be associated with increased microbial growth as seen in this study. Cannibalism and predation of other fish is expected with African catfish and is increased during low stocking density (Haylor, 1991), lack of shelter, and decreased food availability (Hecht & Appelbaum, 1988). Although we had to exclude the catfish growth performance in the Maldeco feed due to a higher initial biomass, the percent weight gain (PWG) was very low at week 4 suggesting manure fertilizing had better performance than commercial fish feed for catfish. The Maldeco feed was not extruded, thus instead of floating on the surface it sank rapidly leaving a narrow window of time for the fish to actually consume it. For this reason along with measuring satiation occurrence, floating feeds are preferred over sinking feeds (Yaqoob, Ali, & Mehmood, 2010). As fish growth (tilapia or catfish) was not statistically improved with the use of fish feed, this suggests that fish feed in its current status may only contribute organic matter and thus act as a fertilizer rather than direct feed. As expected, fish performance was poorest in the cow and pig manure for both tilapia and catfish likely due to decreased microbial production from decreased nutrient inputs (Eghball, Wienhold, Gilley, & Eigenberg, 2002). The SGR for tilapia was previously determined to be 0.69% with demonstrated improvements due to consumption of pond plankton (Chikafumbwa, Costa‐Pierce, Jamu, Kadongola, & Balarin, 1993). Our study used 21 smaller experimental concrete ponds with a stocking density of 2.5 fish per m^2^, which is the common stocking density in earthen ponds in Malawi since aeration is generally not available. Any potential benefits offered by the broiler and quail manure for polyculture of tilapia and catfish should be replicated in a larger growout earthen pond for further validation.

Livestock manure applied to fertilize ponds may benefit the ecosystem by providing macronutrients like Nitrate and Phosphate for supporting photosynthetic microalgae, organic matter as an energy source for heterotrophic bacteria, or living bacteria which can promote pond water stability or a gut probiotic for fish. Water visibility in ponds can be used as a direct indicator of microbial plankton growth or primary productivity (Almazan & Boyd, 1978). Various aspects influence the microbial ecology of the fish gastrointestinal system including diet, trophic level (Liu et al., 2016), habitat, and phylogeny (Tarnecki et al., 2017; Wong et al., 2013). Despite occupying the same environment and being exposed to the same food sources, polycultured tilapia, and catfish shared only a few common fish specific fecal microbes, largely having unique host‐specific microbiomes. Various microbes including *Bacilli* (Cutting, 2011) have been demonstrated to have probiotic potential in the fish gut through antimicrobial production, immunostimulation, and competitive exclusion (Gatesoupe, 1999; Newaj‐Fyzul, Al‐Harbi, & Austin, 2014; Ringø & Gatesoupe, 1998) and since *Bacillus* were prominent microbial members of the bird manures it could be a direct source. Actinobacteria were also highly abundant in manure samples and can have high production rates of secondary metabolites such as antimicrobials and growth promoters for plants (Qin, Xing, Jiang, Xu, & Li, 2011). *Actinobacteria* are also coupled to microalgae production (Das, Ward, & Burke, 2008); thus, bird manure may promote pond phytoplankton production by contributing probiotics to the water column.

Manure fertilizer type influenced the fish pond microbial ecology including the water column and polycultured fish feces, but only a subset of the actual manure associated microbes were detectable in the pond ecosystem. This suggests that manure fertilizer primarily influences ponds through providing unique sources of organic matter and nutrients rather than a direct source of microbes. Many animal gut associated microbes are anaerobic (Dowd et al., 2008), occupying a unique niche (Thompson et al., 2017) and thus may not be capable of surviving in an aerobic water environment. Tilapia did have a higher number of manure associated sOTUs than water at week 4 and catfish feces implying that tilapia gut environments may be most favorable for colonization of manure microbes. Feeding ecology may also driver exposure since tilapia are generally filter feeders, whereas catfish are primarily benthic carnivores. As many of these microbes are common, it is also possible that they are not necessarily transferred from the animal manure but simply represent microbial populations that are commonly found in each environment. It may also suggest that tilapia are more susceptible to pond water microbiology disturbances than catfish which could have important conservation and seafood consumption implications. Both fish species had *Lactobacillus* and *E. coli* sOTUs shared with guinea fowl, layer chicken, and quail manure. Similarly, *Cetobacterium somerae* was shared from guinea fowl and layer manure in both catfish and tilapia. *C. somerae* is a common anaerobic bacteria found in many freshwater fish guts (Tsuchiya, Sakata, & Sugita, 2008), thus our finding implies a potential source of this microbe in the pond system. The presence of *E. coli* or *S. flexneri* in the catfish gut and shared with animal manure samples may be important for human consumption safety as strains of these microbes cause over 1.1 million diarrhea infections per year in children from developing countries worldwide (Jennison & Verma, 2004). Understanding how environmental microbes may influence an aquatic ecosystem such as a fish pond will ultimately depend on the fish species being reared along with other factors. For aquaculture applications, future studies should focus on long‐term growout studies in earthen ponds to validate the enhanced production by broiler chicken and quail manure along with short‐term comparisons across various development stages (fry, fingerling, juvenile, and adult). Conservation studies should not just look at one model fish species but many if environmental degradation is of question. As our results demonstrate how microbial communities influenced by manure treatments can have a beneficial effect on catfish fingerlings, we hypothesize that further optimization could also improve catfish fry production which is currently a limitation in many African countries including Malawi (Chelewani, Kassam, & Chiwanda, 2016).

Food safety is of growing concern worldwide particularly in developed countries where livestock living conditions and cleanliness can have significant influence on consumer preferences. An important concern with meat quality is exposure or presence of antibiotics which freshwater fish ponds have been shown to be potential reservoirs of antibiotic resistance genes (Xiong et al., 2015). One of the primary concerns with growing fish intended for human consumption in manure fertilized ponds is the potential transfer of antibiotics from terrestrial agriculture animals to fish which over time could lead to an increase in antibiotic resistance genes. In our study, Antibiotic resistant genes were highest in manure and water samples, while tilapia had higher amounts than catfish. No tetracycline resistance genes were observed which have been mostly associated with agriculture practices (Gibson et al., 2015). ABC transporters have been primarily associated with Firmicutes (Forsberg et al., 2014), which in our study is associated with manure and fish fecal samples. Actinobacteria were also prevalent in bird manure, water, and fish fecal samples and are generally associated with high antibiotic resistance genes, particularly MFS transporters (Forsberg et al., 2014). Lastly, fluoroquinolone antibiotics have been used to treat Malaria for many decades (Divo, Sartorelli, Patton, & Bia, 1988), but was replaced in 1993 by other drugs (Laufer et al., 2006), although this class of antibiotics is still widely used to treat a variety of gram negative bacterial infections as ciprofloxacin (Makoka et al., 2012). Antibiotics are used broadly in hospital and other rural clinics in Malawi, while agriculture use is not well documented. It is possible that the livestock manure contribute to ARG prevalence in fish ponds, but it is also very likely that the primary source is from the river water irrigated at the site. Tanks that did not receive any animal manure input, but instead were given fish feed, had the same levels of ARGs in the water and fish feces (Figure 5a). This provides evidence for a common source such as the water.

## 5 CONCLUSION

The present study is the first to evaluate the microbial effects of manure‐based fertilization methods in freshwater aquaculture ponds and examine influences on performance of tilapia and catfish grown in polyculture. The objective was to understand the prebiotic or probiotic mechanisms of livestock manure applied as fertilizer in aquaculture ponds. Our results demonstrate that while manure type influences the microbial composition of the water column and fish guts with broiler chicken and quail having a positive influence on fish production, manure microbes are largely undetectable in the pond water and fish feces. This suggests that fertilization with livestock manure has a primarily prebiotic effect on the system by contributing organic matter, macronutrients, and micronutrients which in turn influences the pond microbes. Furthermore, this finding suggests that as microbial transmission from animal manure to fish is low, that human consumption concerns should instead focus on storage and processing safety. While manure samples had the highest amounts of ARGs, few ARGs were detected in the fish fecal samples. In turn, of the few manure associated microbes detected in the fish feces, some were potentially probiotic bacteria and suggest further follow‐up studies to focus on optimizing manure fertilization of hatchery fry to fingerling nursery systems especially for catfish.

## ETHICS APPROVAL

All research conducted on fish was approved by the government of Malawi. The fish were not harmed in any manner. In addition to the pond experiment, three additional catfish were purchased from NAC during a public fish sale. These fish were part of another experiment at NAC and which were harvested from NAC and sold to the community.

## AVAILABILITY OF DATA AND MATERIALS

The 16S, 18S, and metagenomics datasets generated and/or analyzed during the current study are available at Qiita.ucsd.edu server under study ID 10353. In addition, all data have been submitted to EBI: ERP106745.

## COMPETING INTERESTS

The authors declare that they have no competing interests in this section.

## AUTHOR CONTRIBUTIONS

JJM involved with experiment design and carried out experiment in Malawi, performed all microbiome processing, performed amplicon and WGS analysis, and was the primary composer of the manuscript; QZ helped with shotgun metagenomics processing and analysis and assisted in writing; ZX performed the ARG analysis; AA assisted in the 16S and 18S processing and data generation; MN collected, identified, and isolated the 5 zoo plankton isolates used as controls; MS oversaw and helped with carrying out fish culturing experiment; EEA provided guidance on microbiome analyses; HZ provided supervision in carrying out fish culturing experiment, assisted in experimental design, and helped in analysis of fish performance; RK provided supervision for microbiome analyses.

## References

[mbo3716-bib-0001] Almazan, G. , & Boyd, C. E. (1978). An evaluation of Secchi disk visibility for estimating plankton density in fish ponds. Hydrobiologia, 61(3), 205–208. 10.1007/BF00044446

[mbo3716-bib-0002] Amaral‐Zettler, L. A. , McCliment, E. A. , Ducklow, H. W. , & Huse, S. M. (2009). A method for studying Protistan diversity using massively parallel sequencing of V9 hypervariable regions of small‐subunit ribosomal RNA genes. PLoS ONE, 4(7), e6372 10.1371/journal.pone.0006372 19633714 PMC2711349

[mbo3716-bib-0003] Amir, A. , McDonald, D. , Navas‐Molina, J. A. , Kopylova, E. , Morton, J. T. , Zech Xu, Z. , … Knight, R. (2017). Deblur rapidly resolves single‐nucleotide community sequence patterns. MSystems, 2(2), e00191‐16 10.1128/mSystems.00191-16 28289731 PMC5340863

[mbo3716-bib-0004] Anderson, M. J. (2001). A new method for non‐parametric multivariate analysis of variance. Austral Ecology, 26(1), 32–46. 10.1111/j.1442-9993.2001.01070.pp.x

[mbo3716-bib-0005] Appelbaum, S. , & McGeer, J. C. (1998). Effect of diet and light regime on growth and survival of African catfish (*Clarias gariepinus*) larvae and early juveniles. Aquaculture Nutrition, 4(3), 157–164. 10.1046/j.1365-2095.1998.00064.x

[mbo3716-bib-0006] Balcázar, J. L. , de Blas, I. , Ruiz‐Zarzuela, I. , Cunningham, D. , Vendrell, D. , & Múzquiz, J. L. (2006). The role of probiotics in aquaculture. Veterinary Microbiology, 114(3), 173–186. 10.1016/j.vetmic.2006.01.009 16490324

[mbo3716-bib-0007] Benjamini, Y. , & Hochberg, Y. (1995). Controlling the false discovery rate: A practical and powerful approach to multiple testing. Journal of the Royal Statistical Society. Series B (Methodological), 57(1), 289–300.

[mbo3716-bib-0008] Bok, A. H. , & Jongbloed, H. (1984). Growth and production of sharptooth catfish, *Clarias gariepinus* (Pisces: Clariidae), in organically fertilized ponds in the Cape Province, South Africa. Aquaculture, 36(1), 141–155. 10.1016/0044-8486(84)90060-7

[mbo3716-bib-0009] Bolger, A. M. , Lohse, M. , & Usadel, B. (2014). Trimmomatic: A flexible trimmer for Illumina sequence data. Bioinformatics, 30(15), 2114–2120. 10.1093/bioinformatics/btu170 24695404 PMC4103590

[mbo3716-bib-0010] Boyd, C. E. (1982). Water quality management for pond fish culture. Amsterdam, Netherlands: Elsevier Scientific Publishing Co Retrieved from https://www.cabdirect.org/cabdirect/abstract/19821428342

[mbo3716-bib-0011] Cabello, F. C. , Godfrey, H. P. , Tomova, A. , Ivanova, L. , Dölz, H. , Millanao, A. , & Buschmann, A. H. (2013). Antimicrobial use in aquaculture re‐examined: Its relevance to antimicrobial resistance and to animal and human health. Environmental Microbiology, 15(7), 1917–1942. 10.1111/1462-2920.12134 23711078

[mbo3716-bib-0012] Caporaso, J. G. , Kuczynski, J. , Stombaugh, J. , Bittinger, K. , Bushman, F. D. , Costello, E. K. , … Knight, R. (2010). QIIME allows analysis of high‐throughput community sequencing data. Nature Methods, 7(5), 335–336. 10.1038/nmeth.f.303 20383131 PMC3156573

[mbo3716-bib-0013] Caporaso, J. G. , Lauber, C. L. , Walters, W. A. , Berg‐Lyons, D. , Huntley, J. , Fierer, N. , … Knight, R. (2012). Ultra‐high‐throughput microbial community analysis on the Illumina HiSeq and MiSeq platforms. The ISME Journal, 6(8), 1621–1624. 10.1038/ismej.2012.8 22402401 PMC3400413

[mbo3716-bib-0014] Caporaso, J. G. , Lauber, C. L. , Walters, W. A. , Berg‐Lyons, D. , Lozupone, C. A. , Turnbaugh, P. J. , … Knight, R. (2011). Global patterns of 16S rRNA diversity at a depth of millions of sequences per sample. Proceedings of the National Academy of Sciences, 108(Supplement 1), 4516–4522. 10.1073/pnas.1000080107 PMC3063599 20534432

[mbo3716-bib-0015] Chelewani, A. P. , Kassam, D. , & Chiwanda, V. J. M. (2016). Assessment of growth and survival rates of African Catfish (*Clarias gariepinus* BURCHELL 1822) fry fed on soybean milk‐based diets. International Journal of Aquaculture, 6(7), 1–10. Retrieved from http://biopublisher.ca/index.php/ija/article/view/2813

[mbo3716-bib-0016] Chikafumbwa, F. J. K. T. , Costa‐Pierce, B. A. , Jamu, D. M. , Kadongola, W. K. , & Balarin, J. D. (1993). Investigations on the use of on‐farm resources as pond inputs to culture *Tilapia rendalli* and *Oreochromis shiranus* on smallholder farms in rural Malawi. Aquaculture, 117(3), 261–271. 10.1016/0044-8486(93)90324-R

[mbo3716-bib-0017] Cutting, S. M. (2011). Bacillus probiotics. Food Microbiology, 28(2), 214–220. 10.1016/j.fm.2010.03.007 21315976

[mbo3716-bib-0018] Das, S. , Ward, L. R. , & Burke, C. (2008). Prospects of using marine actinobacteria as probiotics in aquaculture. Applied Microbiology and Biotechnology, 81(3), 419–429. 10.1007/s00253-008-1731-8 18841358

[mbo3716-bib-0019] Davies, J. , & Davies, D. (2010). Origins and evolution of antibiotic resistance. Microbiology and Molecular Biology Reviews, 74(3), 417–433. 10.1128/MMBR.00016-10 20805405 PMC2937522

[mbo3716-bib-0020] Divo, A. A. , Sartorelli, A. C. , Patton, C. L. , & Bia, F. J. (1988). Activity of fluoroquinolone antibiotics against *Plasmodium falciparum* in vitro. Antimicrobial Agents and Chemotherapy, 32(8), 1182–1186. 10.1128/AAC.32.8.1182 2847647 PMC172373

[mbo3716-bib-0021] Dowd, S. E. , Callaway, T. R. , Wolcott, R. D. , Sun, Y. , McKeehan, T. , Hagevoort, R. G. , & Edrington, T. S. (2008). Evaluation of the bacterial diversity in the feces of cattle using 16S rDNA bacterial tag‐encoded FLX amplicon pyrosequencing (bTEFAP). BMC Microbiology, 8, 125 10.1186/1471-2180-8-125 18652685 PMC2515157

[mbo3716-bib-0022] Eghball, B. , Wienhold, B. J. , Gilley, J. E. , & Eigenberg, R. A. (2002). Mineralization of manure nutrients. Journal of Soil and Water Conservation, 57(6), 470–473.

[mbo3716-bib-0023] Finn, R. D. , Clements, J. , & Eddy, S. R. (2011). HMMER web server: Interactive sequence similarity searching. Nucleic Acids Research, 39(suppl_2), W29–W37. 10.1093/nar/gkr367 21593126 PMC3125773

[mbo3716-bib-0024] Forsberg, K. J. , Patel, S. , Gibson, M. K. , Lauber, C. L. , Knight, R. , Fierer, N. , & Dantas, G. (2014). Bacterial phylogeny structures soil resistomes across habitats. Nature, 509(7502), 612–616. 10.1038/nature13377 24847883 PMC4079543

[mbo3716-bib-0025] Froese, R. (2006). Cube law, condition factor and weight‐length relationships: History, meta‐analysis and recommendations. Journal of Applied Ichthyology, 22, 241–253. 10.1111/j.1439-0426.2006.00805.x

[mbo3716-bib-0026] FULTON, T. (1904). The rate of growth of fishes. *Twenty‐Second Annual Report*, 141–241.

[mbo3716-bib-0027] Gatesoupe, F. J. (1999). The use of probiotics in aquaculture. Aquaculture, 180(1), 147–165. 10.1016/S0044-8486(99)00187-8

[mbo3716-bib-0028] Ghanbari, M. , Kneifel, W. , & Domig, K. J. (2015). A new view of the fish gut microbiome: Advances from next‐generation sequencing. Aquaculture, 448, 464–475. 10.1016/j.aquaculture.2015.06.033

[mbo3716-bib-0029] Gibson, M. K. , Forsberg, K. J. , & Dantas, G. (2015). Improved annotation of antibiotic resistance determinants reveals microbial resistomes cluster by ecology. The ISME Journal, 9(1), 207–216. 10.1038/ismej.2014.106 25003965 PMC4274418

[mbo3716-bib-0030] Haylor, G. S. (1991). Controlled hatchery production of *Clarias gariepinus* (Burchell 1822): Growth and survival of fry at high stocking density. Aquaculture Research, 22(4), 405–422. 10.1111/j.1365-2109.1991.tb00754.x

[mbo3716-bib-0031] Hecht, T. , & Appelbaum, S. (1988). Observations on intraspecific aggression and coeval sibling cannibalism by larval and juvenile *Claias gariepinus* (Clariidae: Pisces) under controlled conditions. Journal of Zoology, 214(1), 21–44. 10.1111/j.1469-7998.1988.tb04984.x

[mbo3716-bib-0032] Hetherington, J. B. , Wiethoelter, A. K. , Negin, J. , & Mor, S. M. (2017). Livestock ownership, animal source foods and child nutritional outcomes in seven rural village clusters in Sub‐Saharan Africa. Agriculture & Food Security, 6, 9 10.1186/s40066-016-0079-z

[mbo3716-bib-0033] Heuer, H. , Schmitt, H. , & Smalla, K. (2011). Antibiotic resistance gene spread due to manure application on agricultural fields. Current Opinion in Microbiology, 14(3), 236–243. 10.1016/j.mib.2011.04.009 21546307

[mbo3716-bib-0034] Hofer, R. (1998). Morphological adaptations of the digestive tract of tropical cyprinids and cichlids to diet. Journal of Fish Biology, 33(3), 399–408. 10.1111/j.1095-8649.1988.tb05481.x

[mbo3716-bib-0035] Hopkins, K. D. (1992). Reporting fish growth: A review of the Basics1. Journal of the World Aquaculture Society, 23(3), 173–179. 10.1111/j.1749-7345.1992.tb00766.x

[mbo3716-bib-0036] Hossain, M. A. R. , Beveridge, M. C. M. , & Haylor, G. S. (1998). The effects of density, light and shelter on the growth and survival of African catfish (*Clarias gariepinus* Burchell, 1822) fingerlings. Aquaculture, 160(3), 251–258. 10.1016/S0044-8486(97)00250-0

[mbo3716-bib-0037] Irvine, K. , & Waya, R. (1999). Spatial and temporal patterns of zooplankton standing biomass and production in Lake Malawi. Hydrobiologia, 407, 191–205. 10.1023/A:1003711306243

[mbo3716-bib-0038] Jamu, D. , Banda, M. , Njaya, F. , & Hecky, R. E. (2011). Challenges to sustainable management of the lakes of Malawi. Journal of Great Lakes Research, 37, 3–14. 10.1016/j.jglr.2010.11.017

[mbo3716-bib-0039] Jennison, A. V. , & Verma, N. K. (2004). *Shigella flexneri* infection: Pathogenesis and vaccine development. FEMS Microbiology Reviews, 28(1), 43–58. 10.1016/j.femsre.2003.07.002 14975529

[mbo3716-bib-0040] Kang'ombe, J. , Brown, J. A. , & Halfyard, L. C. (2006). Effect of using different types of organic animal manure on plankton abundance, and on growth and survival of *Tilapia rendalli* (Boulenger) in ponds. Aquaculture Research, 37(13), 1360–1371. 10.1111/j.1365-2109.2006.01569.x

[mbo3716-bib-0041] Kawarazuka, N. , & Béné, C. (2011). The potential role of small fish species in improving micronutrient deficiencies in developing countries: Building evidence. Public Health Nutrition, 14(11), 1927–1938. 10.1017/S1368980011000814 21729489

[mbo3716-bib-0042] Langdon, W. B. (2015). Performance of genetic programming optimised Bowtie2 on genome comparison and analytic testing (GCAT) benchmarks. BioData Mining, 8, 1 10.1186/s13040-014-0034-0 25621011 PMC4304608

[mbo3716-bib-0043] Laufer, M. K. , Thesing, P. C. , Eddington, N. D. , Masonga, R. , Dzinjalamala, F. K. , Takala, S. L. , … Plowe, C. V. (2006). Return of chloroquine antimalarial efficacy in Malawi. New England Journal of Medicine, 355(19), 1959–1966. 10.1056/NEJMoa062032 17093247

[mbo3716-bib-0044] Lazado, C. C. , & Caipang, C. M. A. (2014). Mucosal immunity and probiotics in fish. Fish & Shellfish Immunology, 39(1), 78–89. 10.1016/j.fsi.2014.04.015 24795079

[mbo3716-bib-0045] Liu, H. , Guo, X. , Gooneratne, R. , Lai, R. , Zeng, C. , Zhan, F. , & Wang, W. (2016). The gut microbiome and degradation enzyme activity of wild freshwater fishes influenced by their trophic levels. Scientific Reports, 6, 24340 10.1038/srep24340 27072196 PMC4829839

[mbo3716-bib-0046] Lozupone, C. , & Knight, R. (2005). UniFrac: A new phylogenetic method for comparing microbial communities. Applied and Environmental Microbiology, 71(12), 8228–8235. 10.1128/AEM.71.12.8228-8235.2005 16332807 PMC1317376

[mbo3716-bib-0047] Lugert, V. , Thaller, G. , Tetens, J. , Schulz, C. , & Krieter, J. (2016). A review on fish growth calculation: Multiple functions in fish production and their specific application. Reviews in Aquaculture, 8(1), 30–42. 10.1111/raq.12071

[mbo3716-bib-0048] Makoka, M. H. , Miller, W. C. , Hoffman, I. F. , Cholera, R. , Gilligan, P. H. , Kamwendo, D. , … Hosseinipour, M. C. (2012). Bacterial infections in Lilongwe, Malawi: Aetiology and antibiotic resistance. BMC Infectious Diseases, 12, 67 10.1186/1471-2334-12-67 22436174 PMC3342226

[mbo3716-bib-0049] Mataka, L. , & Kang'ombe, J. (2007). Effect of substitution of maize bran with chicken manure in semi‐intensive pond culture of *Tilapia rendalli* (Boulenger). Aquaculture Research, 38(9), 940–946. Retrieved from http://agris.fao.org/agris-search/search.do?recordID=US201300783629

[mbo3716-bib-0050] Moav, R. , & Wohlfarth, G. W. (1974). Magnification through competition of genetic differences in yield capacity in carp^1^ . Heredity, 33(2), 181–202. 10.1038/hdy.1974.86 4531427

[mbo3716-bib-0051] Moffitt, C. M. , & Cajas‐Cano, L. (2014). Blue growth: The 2014 FAO state of world fisheries and aquaculture. Fisheries, 39(11), 552–553. 10.1080/03632415.2014.966265

[mbo3716-bib-0052] Munthali, S. M. (1997). Dwindling food‐fish species and fishers’ preference: Problems of conserving Lake Malawi's biodiversity. Biodiversity & Conservation, 6(2), 253–261. 10.1023/A:1018396120503

[mbo3716-bib-0053] Neori, A. (2011). “Green water” microalgae: The leading sector in world aquaculture. Journal of Applied Phycology, 23(1), 143–149. 10.1007/s10811-010-9531-9

[mbo3716-bib-0054] Newaj‐Fyzul, A. , Al‐Harbi, A. H. , & Austin, B. (2014). Review: Developments in the use of probiotics for disease control in aquaculture. Aquaculture, 431, 1–11. 10.1016/j.aquaculture.2013.08.026

[mbo3716-bib-0055] Prein, M. (2002). Integration of aquaculture into crop–animal systems in Asia. Agricultural Systems, 71(1), 127–146. 10.1016/S0308-521X(01)00040-3

[mbo3716-bib-0056] Qin, S. , Xing, K. , Jiang, J.‐H. , Xu, L.‐H. , & Li, W.‐J. (2011). Biodiversity, bioactive natural products and biotechnological potential of plant‐associated endophytic actinobacteria. Applied Microbiology and Biotechnology, 89(3), 457–473. 10.1007/s00253-010-2923-6 20941490

[mbo3716-bib-0057] Rho, M. , Tang, H. , & Ye, Y. (2010). FragGeneScan: Predicting genes in short and error‐prone reads. Nucleic Acids Research, 38(20), e191–e191. 10.1093/nar/gkq747 20805240 PMC2978382

[mbo3716-bib-0058] Ringø, E. , & Gatesoupe, F.‐J. (1998). Lactic acid bacteria in fish: A review. Aquaculture, 160(3), 177–203. 10.1016/S0044-8486(97)00299-8

[mbo3716-bib-0059] Russell, A. J. M. , Grötz, P. A. , Kriesemer, S. K. , & Pemsl, D. E. (2008). Country case study: Development and status of freshwater aquaculture in Malawi. Penang, Malaysia: WorldFish.

[mbo3716-bib-0060] Sapkota, A. , Sapkota, A. R. , Kucharski, M. , Burke, J. , McKenzie, S. , Walker, P. , & Lawrence, R. (2008). Aquaculture practices and potential human health risks: Current knowledge and future priorities. Environment International, 34(8), 1215–1226. 10.1016/j.envint.2008.04.009 18565584

[mbo3716-bib-0061] Snoeks, J. (2000). How well known is the ichthyodiversity of the large East African lakes? Advances in Ecological Research, 31, 17–38. 10.1016/S0065-2504(00)31005-4

[mbo3716-bib-0062] Su, J.‐Q. , Cui, L. , Chen, Q.‐L. , An, X.‐L. , & Zhu, Y.‐G. (2017). Application of genomic technologies to measure and monitor antibiotic resistance in animals. Annals of the New York Academy of Sciences, 1388(1), 121–135. 10.1111/nyas.13296 27997690

[mbo3716-bib-0063] Tarnecki, A. M. , Burgos, F. A. , Ray, C. L. , & Arias, C. R. (2017). Fish intestinal microbiome: Diversity and symbiosis unraveled by metagenomics. Journal of Applied Microbiology, 123(1), 2–17. 10.1111/jam.13415 28176435

[mbo3716-bib-0064] Thanner, S. , Drissner, D. , & Walsh, F. (2016). Antimicrobial resistance in agriculture. MBio, 7(2), e02227‐15 10.1128/mBio.02227-15 27094336 PMC4850276

[mbo3716-bib-0065] Thompson, L. R. , Sanders, J. G. , McDonald, D. , Amir, A. , Ladau, J. , Locey, K. J. , … Zhao, H. (2017). A communal catalogue reveals Earth's multiscale microbial diversity. Nature, 551(7681), 457–463. 10.1038/nature24621 29088705 PMC6192678

[mbo3716-bib-0066] Tsuchiya, C. , Sakata, T. , & Sugita, H. (2008). Novel ecological niche of *Cetobacterium somerae*, an anaerobic bacterium in the intestinal tracts of freshwater fish. Letters in Applied Microbiology, 46(1), 43–48. 10.1111/j.1472-765X.2007.02258.x 17944860

[mbo3716-bib-0067] Vázquez‐Baeza, Y. , Pirrung, M. , Gonzalez, A. , & Knight, R. (2013). EMPeror: A tool for visualizing high‐throughput microbial community data. GigaScience, 2, 16 10.1186/2047-217X-2-16 24280061 PMC4076506

[mbo3716-bib-0068] Walters, W. , Hyde, E. R. , Berg‐Lyons, D. , Ackermann, G. , Humphrey, G. , Parada, A. , … Knight, R. (2016). Improved bacterial 16S rRNA gene (V4 and V4‐5) and Fungal internal transcribed spacer marker gene primers for microbial community surveys. MSystems, 1(1), e00009‐15 10.1128/mSystems.00009-15 PMC5069754 27822518

[mbo3716-bib-0069] Wang, A. R. , Ran, C. , Ringø, E. , & Zhou, Z. G. (2017). Progress in fish gastrointestinal microbiota research. Reviews in Aquaculture, 10(3), 626–640. 10.1111/raq.12191

[mbo3716-bib-0070] Willoughby, N. G. , & Tweddle, D. (1978). The ecology of the catfish *Clarias gariepinus* and *Clarias ngamensis* in the Shire Valley, Malawi. Journal of Zoology, 186(4), 507–534. 10.1111/j.1469-7998.1978.tb03936.x

[mbo3716-bib-0071] Wong, S. , Waldrop, T. , Summerfelt, S. , Davidson, J. , Barrows, F. , Kenney, P. B. , … Good, C. (2013). Aquacultured rainbow trout (*Oncorhynchus mykiss*) possess a large core intestinal microbiota that is resistant to variation in diet and rearing density. Applied and Environmental Microbiology, 79(16), 4974–4984. 10.1128/AEM.00924-13 23770898 PMC3754725

[mbo3716-bib-0072] Wood, D. E. , & Salzberg, S. L. (2014). Kraken: Ultrafast metagenomic sequence classification using exact alignments. Genome Biology, 15, R46 10.1186/gb-2014-15-3-r46 24580807 PMC4053813

[mbo3716-bib-0073] Xiong, W. , Sun, Y. , Zhang, T. , Ding, X. , Li, Y. , Wang, M. , & Zeng, Z. (2015). Antibiotics, antibiotic resistance genes, and bacterial community composition in fresh water aquaculture environment in China. Microbial Ecology, 70(2), 425–432. 10.1007/s00248-015-0583-x 25753824

[mbo3716-bib-0074] Yaqoob, M , Ali, M. R. , & Mehmood, S. (2010). Comparison of growth performance of major and Chinese carps fed on floating and sinking pelleted supplementary feeds in ponds. Pakistan Journal of Zoology; Lahore, 42(6), 765–769. Retrieved from https://search.proquest.com/docview/920120350/citation/DEC1CA0FFE524BC5PQ/1

[mbo3716-bib-0075] Zhao, W. , & Shen, H. (2016). A statistical analysis of China's fisheries in the 12th five‐year period. Aquaculture and Fisheries, 1, 41–49. 10.1016/j.aaf.2016.11.001

[mbo3716-bib-0076] Zhu, Y.‐G. , Johnson, T. A. , Su, J.‐Q. , Qiao, M. , Guo, G.‐X. , Stedtfeld, R. D. , … Tiedje, J. M. (2013). Diverse and abundant antibiotic resistance genes in Chinese swine farms. Proceedings of the National Academy of Sciences, 110(9), 3435–3440. 10.1073/pnas.1222743110 PMC3587239 23401528

