# Supplementary material for: Microbial effects of livestock manure fertilization on freshwater aquaculture ponds rearing tilapia (Oreochromis shiranus) and North African catfish (Clarias gariepinus)
Source: Microbiologyopen. 2018 Aug 31;7(6):e00716. doi: 10.1002/mbo3.716 (PMC6291788; doi:10.1002/mbo3.716)
Supplement: Supplementary file 1 [file MBO3-7-e00716-s001.pdf]

SUPPLEMENTAL FIGURE LEGENDS

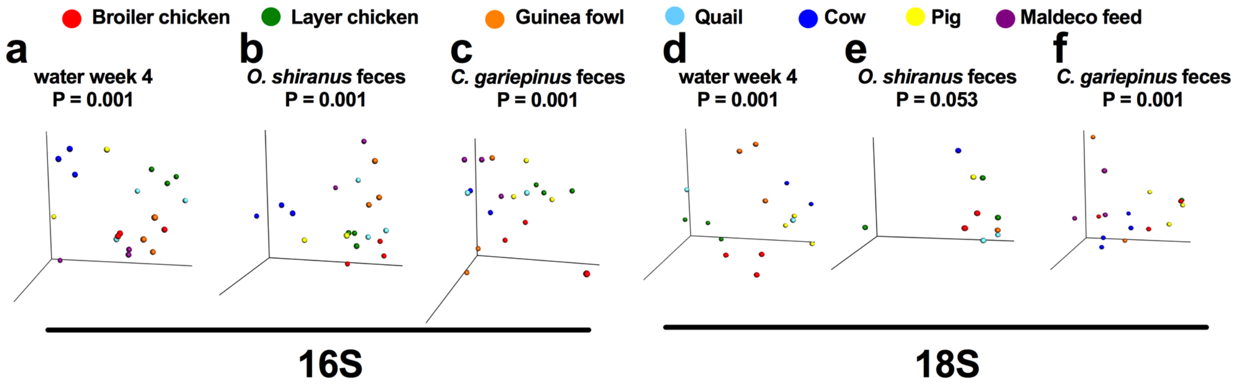

Additional File 1. Principle coordinates analysis (PCoA) plot of microbial beta diversity as measured by unweighted UniFrac distances and highlighted by fertilizer type. 16S rRNA PCoA of (a) water column at week 4, (b) *O. shiranus* feces, and (c) *C. gariepinus* feces. 18S rRNA PCoA of (d) water column at week 4, (e) *O. shiranus* feces, (f) *C. gariepinus* feces.

a

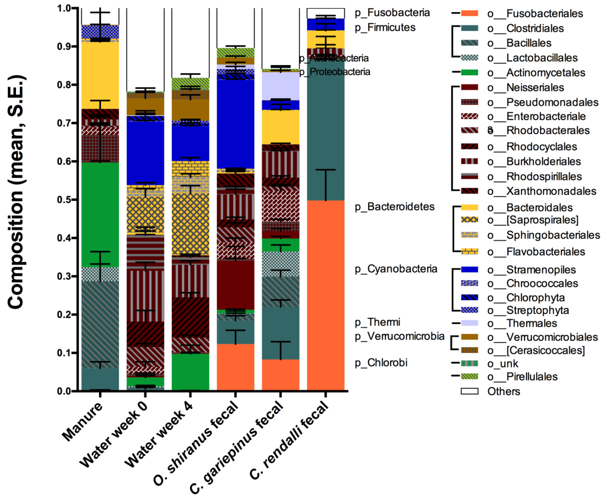

b

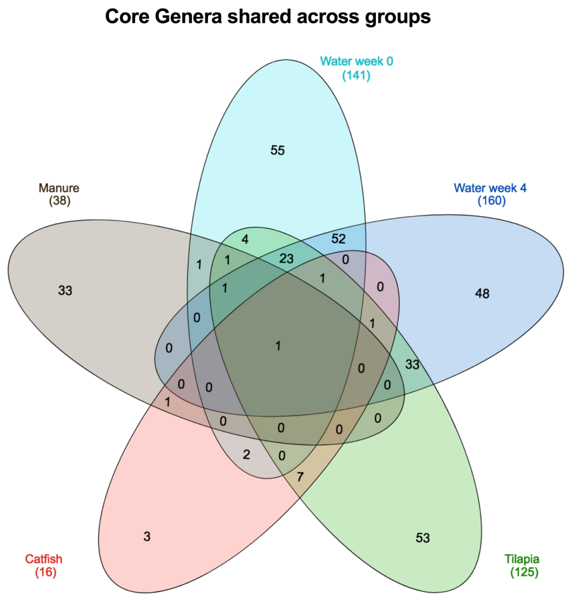

Additional File 2. 16S microbiome summary. (a) Microbial composition (mean, S.E.) across sample types. (b) Core microbes present in at least 75% of samples from an environment type compared to other environment types.

a

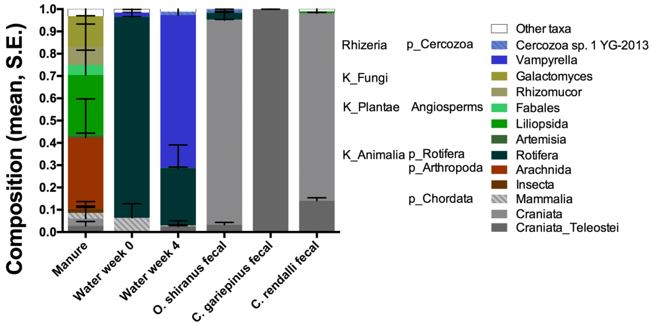

b

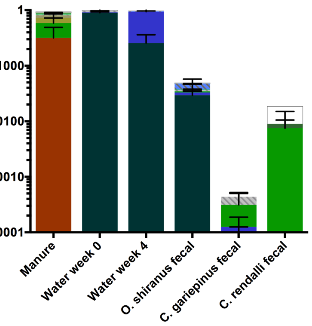

c

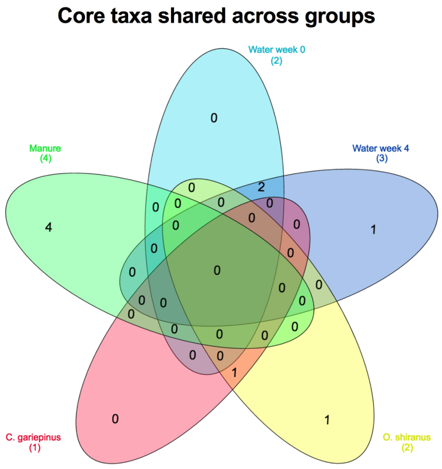

Additional File 3. 18S microbiome summary. (a) Total and (b) host depleted 18S microbial composition (mean, S.E.) across sample types. (c) Core microbes present in at least 75% of samples from an environment type compared to other environment types.

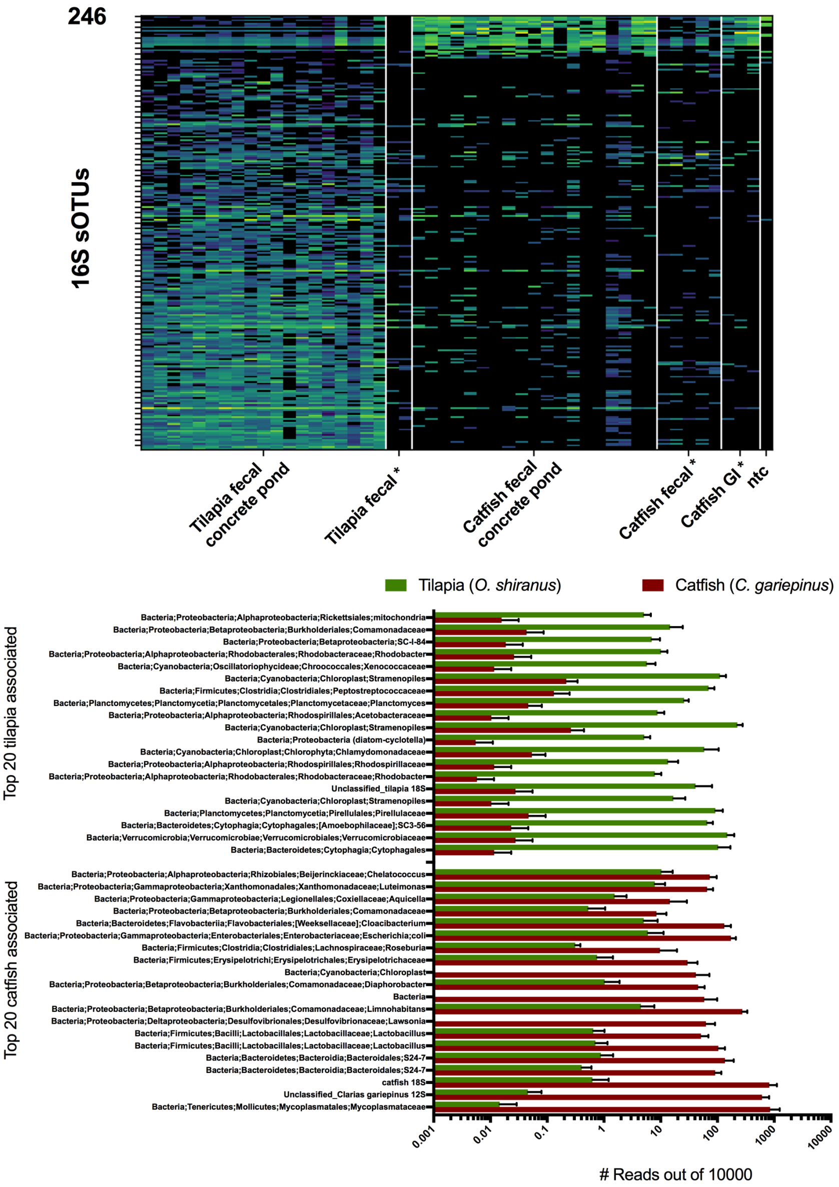

Additional File 4. Differential abundance 16S sOTUs of tilapia (*O. shiranus*) vs. catfish (*C. garpiepinus*). (a) 246 total 16S sOTUs differentially abundant between tilapia and catfish. (b) Top 20 tilapia and catfish associated 16S sOTUs as ranked by difference in ratio.

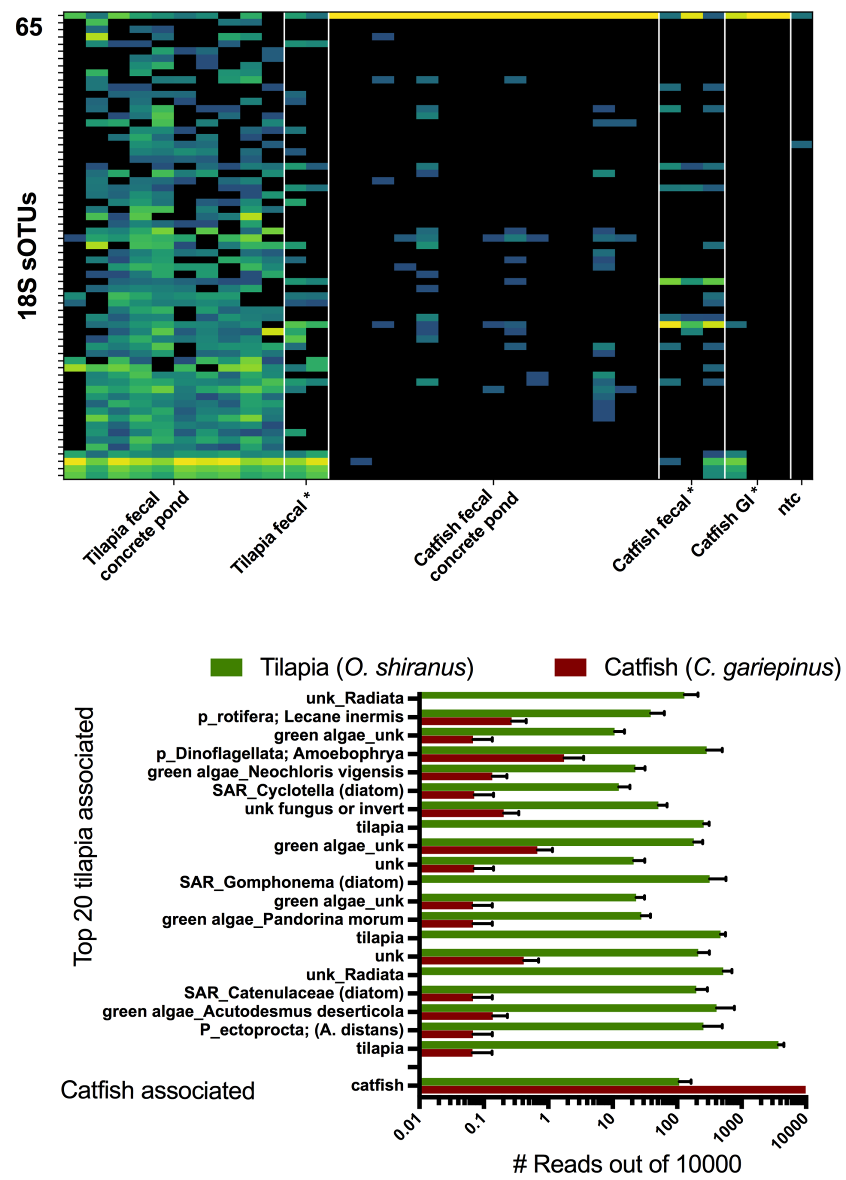

Additional File 5. Differential abundance 18S sOTUs of tilapia (*O. shiranus*) vs. catfish (*C. garpiepinus*). (a) 65 total 18S sOTUs differentially abundant between tilapia and catfish. (b) Top 20 tilapia and 1 catfish associated 18S sOTUs as ranked by difference in ratio.

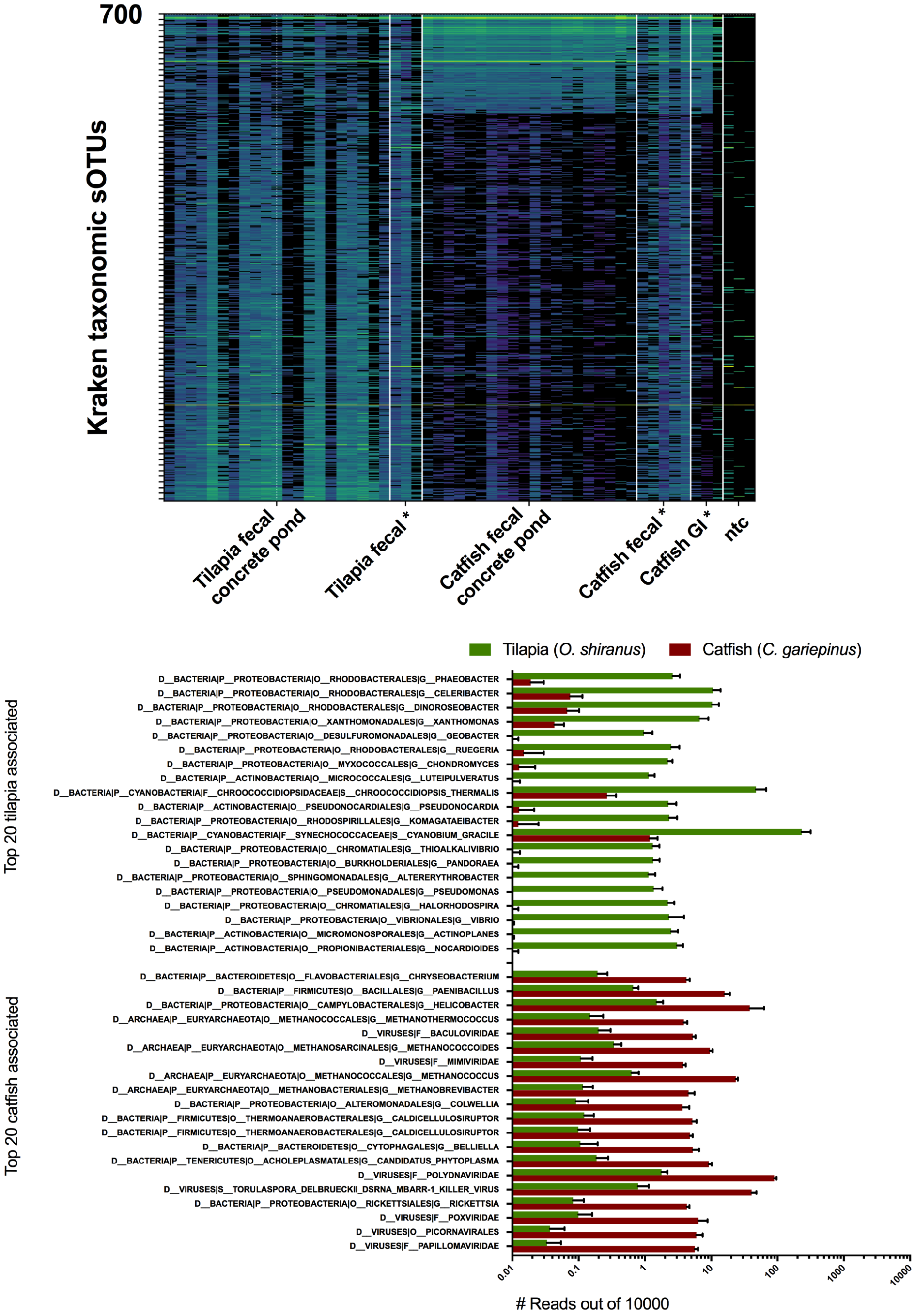

Additional File 6. Differential abundance WGSS taxonomic sOTUs of tilapia (*O. shiranus*) vs. catfish (*C. garpiepinus*). (a) 700 total WGSS sOTUs differentially abundant between tilapia and catfish. (b) Top 20 tilapia and catfish associated WGSS sOTUs as ranked by difference in ratio.
